# Late onset presentation of nephrocalcinosis and nephrolithiasis in association with a heterozygous *CYP24A1* pathogenic variant

**DOI:** 10.1007/s44162-025-00116-8

**Published:** 2025-09-03

**Authors:** Marwa Abouzeina, Paul Mead, Rhema Okpongete, Holly Mabillard, Robert Geraghty, John A. Sayer

**Affiliations:** 1 North Cumbria Integrated Care NHS Trust, Cumberland Infirmary, Carlisle, UK; 2 Faculty of Medical Sciences, Biosciences Institute, Newcastle University, Newcastle Upon Tyne, UK; 3 Renal Services, Newcastle Upon Tyne NHS Foundation Trust, Newcastle Upon Tyne, UK; 4 National Institute for Health Research, Newcastle Biomedical Research Centre, Newcastle Upon Tyne, UK

**Keywords:** Vitamin D, CYP24A1, Nephrocalcinosis, Nephrolithiasis, Hypercalciuria

## Abstract

*CYP24A1* is gene that encodes one of the cytochrome P450 superfamily enzymes involved in the breakdown of 1,25-dihydroxyvitamin D3. Genetic variants in *CYP24A1* lead to a range of phenotypical and biochemical presentations, including idiopathic infantile hypercalcemia, elevated concentrations of 1,25 dihydroxy vitamin D, adult onset nephrocalcinosis, hypercalciuria, hypercalcemia and nephrolithiasis. Here we present an adult female, aged 68 years of age who presented with intermittent abdominal pain, with a past medical history of hypertension. There was a history of oral vitamin D supplementation, however patient denied tanning bed use. There was a family history of kidney stones, with her mother having recurrent kidney stones. Investigations revealed normal serum calcium and total vitamin D levels but evidence of hypercalciuria. Abdominal imaging revealed bilateral nephrocalcinosis. A genetic screen revealed a heterozygous pathogenic variant in *CYP24A1*. She was managed with stopping vitamin D supplements and encouraging a high fluid intake and initiation of a thiazide diuretic which led to a normalisation of urinary calcium levels. The case exemplifies late onset genetic disease secondary to *CYP24A1* loss of function, likely triggered by excessive vitamin D supplementation.

## Introduction

*CYP24A1* encodes the cytochrome P450 enzyme 24-hydroxylase, a multi-catalytic enzyme responsible for inactivation of 1,25(OH)2D3 into water soluble calcitroic acid [13]. The biochemical hallmark of *CYP24A1* variants is typically a persistently increased 1,25-dihydroxy vitamin D3 level, with often an elevation of total Vitamin D levels. This may lead to elevated serum calcium values as well as hypercalciuria and nephrolithiasis and nephrocalcinosis [12]. There may be childhood presentations, especially with biallelic variants in *CYP24A1* leading to idiopathic infantile hypercalcaemia, which can lead to death [4, 16]. In biallelic disease, adult presentations may occur also, often triggered by pregnancy [9] or exposure to vitamin D. Patients with a single (monoallelic) *CYP24A1* variants tend to present with kidney stone disease and less frequently with nephrocalcinosis and symptomatic hyercalcaemia [3]. Variants in *CYP24A1* are rare but they have a distinct and recognisable biochemical phenotype, and patients may present at any age with nephrocalcinosis and kidney stone formation [6, 20, 21]. Even individuals with monoallelic variants in *CYP24A1* often display distinct biochemical phenotypes [2]. With the increase in the number of adults taking vitamin D supplements (often in high doses and hidden within foods or other supplements), biochemical and clinical phenotypes may be revealed later in life. Thus, *CYP24A1* variants may be identified in older patients with a subclinical course or late manifestation of disease [12].

## Case report

**Fig. 1:**
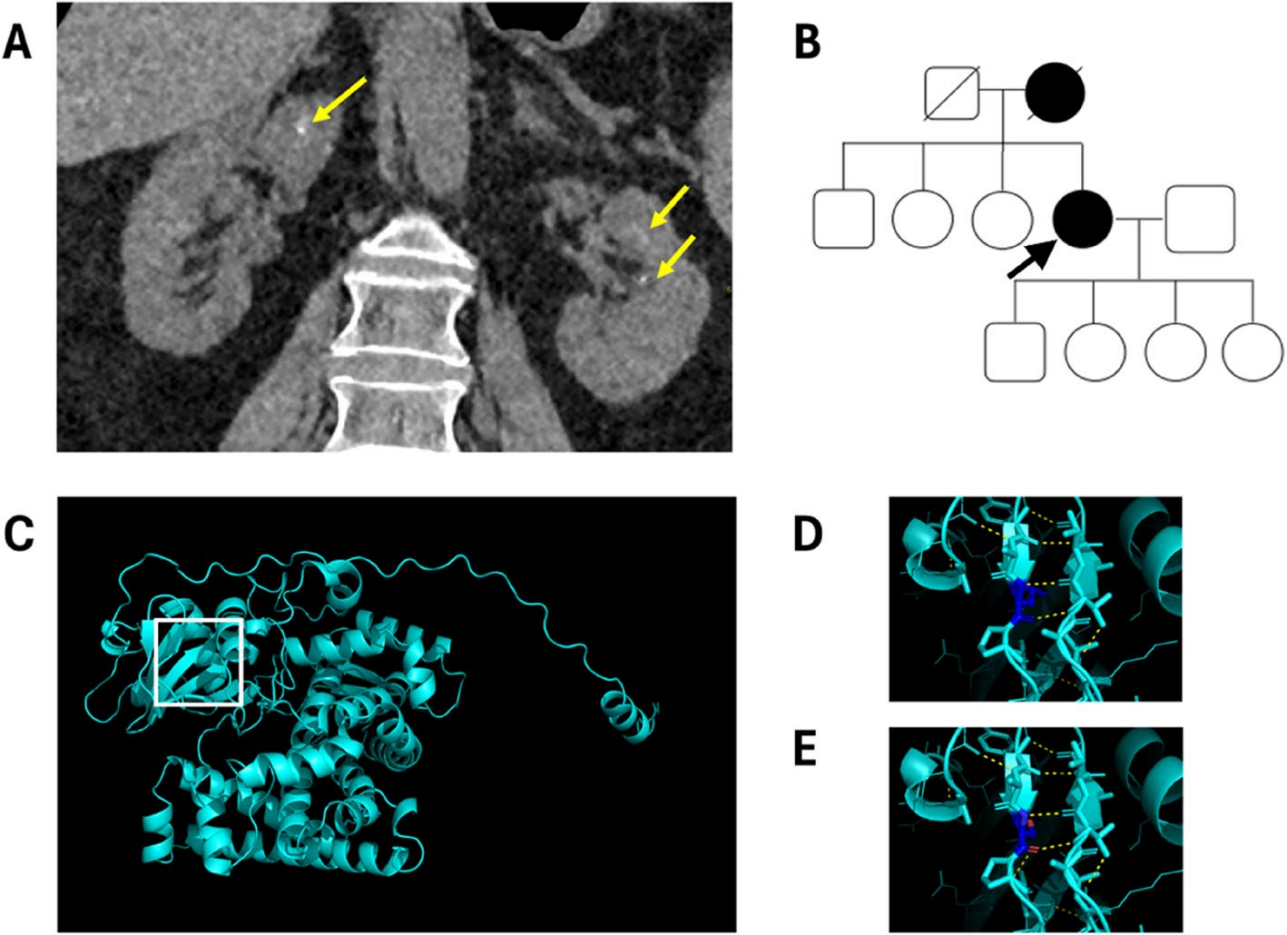
Presentation and investigation of *CYP24A1* related disease. **A** Bilateral mild nephrocalcinosis / nephrolithiasis shown on CT scan (arrowed) in proband. **B** A pedigree diagram showing an autosomal dominant pattern of disease. Shaded symbols affected with kidney stone disease. **C** CYP24A1 amino acid sequence and predicted protein structure downloaded from Alphafold. **D** Predicted interactions for Leucine at position 409 (wild type). **E** Predicted interactions for Serine at position 409—as identified in proband. Note there is an additional predicted interaction between Serine 409 and the adjacent Proline at position 410

A 68 year old female presented with a history of intermittent abdominal pain in her left upper quadrant. She had a past surgical history of a laparoscopic cholecystectomy and a past medical history of controlled hypertension. She was taking amlodipine 10mg once a day for hypertension and oral Cholecalciferol 10 mcg/day (400 IU/day). Blood tests showed normal full blood count, kidney and liver function, normal adjusted serum calcium 2.34 mmol/L (normal range (NR) 2.1–2.6), serum phosphate 1.13 mmol/L (NR 08.-1.5) PTH 4.2 pmol/L (NR 1.6–6.9). Vitamin D levels showed 25-hydroxy vitamin D 45 nmol/L (50–150) and 1,25-dihydroxy Vitamin D 98 pmol/L (NR 20–120). 24,25-dihydroxyvitamin D levels were unavailable. A multiple myeloma screen was negative. An abdominal ultrasound scan was suggestive of nephrocalcinosis and a computed tomography (CT) kidney scan showed nephrocalcinosis (Fig. 1). A 24 h urine collection (2.49 L) showed an elevated urinary calcium (9.1 mmol/24h, NR 2.5–7.5). Other 24 h urine biochemistry values were normal (uric acid 3.0 mmol/24 h (NR < 4.0), cystine 52 μmol/L (NR 0–186), oxalate 0.25 mmol/24 h (0.04–0.32), citrate 3.04 mmol/24 h (1.60–4.50)). The patient was given dietary advice and her anti-hypertensive medication was switched from amlodipine to a thiazide diuretic, as an adjunct to lower urinary calcium levels.

A genetic screen (NHS Genomic Medicine R198 Renal Tubulopathies diagnostic testing panel) revealed a pathogenic heterozygous variant in *CYP24A1* (NM_000782.5: c.1226T > C; p.(Leu409Ser) (Table 1). This prompted a review of her family history which revealed that her mother also had a history of recurrent kidney stones in adult life (Fig. 1). She was managed with the advice to stop taking vitamin D supplements, avoid sunbathing and tanning beds and maintain a high fluid intake. The thiazide diuretic was continued and led to a normalisation of 24 h urine calcium levels. The repeat 24 h urine calcium on thiazide was lower and in the normal range (4.1 mmol/24 (NR 2.5–7.5)) whilst the serum calcium remained in the normal range. Genetic screening of at-risk family revealed normal genotypes for her unaffected siblings.

**Table 1:** Molecular genetic results

Genetic variant	ACMG classification	In vitro assays	gnomAD allele frequency	References
*CYP24A1* NM_000782.5 Heterozygous c.1226T > C; p.(Leu409Ser) (rs6068812)	Pathogenic	Reduced 24-hydroxylase activity [14, 16]	0.001745	[6, 11, 16]

## Discussion

Although disease causing variants in *CYP24A1* are rare, there is growing evidence that disease phenotypes, especially in monoallelic patients, may include late adult presentations of kidney stone disease and nephrocalcinosis [7, 20, 21]. Consideration of *CYP24A1* variants a cause of kidney stone disease should be given and a familial pattern of stone formation should be looked for. The recognition that 40–45% of patients with idiopathic hypercalciuria have at least one relative with nephrolithiasis implicates a genetic predisposition in many cases.^5^

In view of the greatly variable phenotype of the disease, especially in its monogenic form [3, 19], it is noteworthy that many patients, such as the proband presented here, may experience their first clinical complications and diagnosis only in adulthood.^4^ About 30% of the *CYP24A1* cases published so far manifest or are diagnosed in adolescence or adulthood. The precise extent each of these variants result in changes of enzyme activity and thereby contribute to disease severity is not known. Genotyping of patients with familial kidney stone disease is valuable as life-time risk can be reduced by relatively simple dietary restrictions, including avoiding high amounts of calcium and Vitamin D. Indeed, genetic testing for high-risk kidney stone formers has now been incorporated into the latest European Association of Urology guidelines [17]. Both monoallelic and biallelic variants in *CYP24A1* disrupt vitamin D metabolism and elevate risk of raised serum calcium but there is a clear gene dose effect (Table 2). Biallelic changes lead to more severe, early onset disease and higher long term complications. In contrast, monoallelic disease is milder, and is often triggered by factors such as use of tanning beds, vitamin D supplementation and pregnancy, which increases vitamin D activation and calcium absorption [9]. It is noteworthy that the dose or oral vitamin D supplementation in this case was low at just 400 IU Cholecalciferol per day. Typically, much higher maintenance doses (800–1000 IU) are given to patients who are at risk of vitamin D deficiency and general adult multivitamins may contain 600–800 IU. Thus, as seen in this case, even routine low doses may act as triggers for stone disease and hypercalcaemia in patients with *CYP24A1* variants.

**Table 2:** Compare and contrasting presentation, outcomes and treatment of monoallelic versus biallelic CYP24A1-associated disease

	Biallelic* CYP24A1*	Monoallelic *CYP24A1*
Presentation	Infancy and childhood. Often present with idiopathic infantile hypercalcaemia, which can be life-threatening Hypercalcaemia ccan manifest during pregnancy or later life with hypervitaminosis-D. Often severe biochemical features including raised 1,25 dihydroxy vitamin D	Milder more variable phenotype. May be asymptomatic or develop kidney stones, nephrocalcinosis or sporadic hypercalcaemia—sometimes triggered by vitamin D supplementation or other stressors. Biochemical features such as elevated 1,25 dihydroxy vitamin D are less severe
Outcomes	Higher risk of nephrocalcinosis, recurrent kidney stone disease, chronic kidney disease and misdiagnosis (e.g. cystic kidney disease)	Elevated risk compared to unaffected individuals for kidney stone disease and symptomatic hypercalcaemia
Treatment and management	Stop vitamin D supplementation, maintain low-calcium diet, ensure hydration. For severe cases, iv fluids acutely and P450 enzyme inhibitors for longer term treatment	Stop vitamin D supplementation, ensure hydration. Routine monitoring unless provoked (e.g. by vitamin D exposure or pregnancy)

Since the content of calcium or vitamin D can be surprisingly high in particular diets, we would emphasize the necessity of a professional dietary consultation. Natural sources of vitamin D would include cod liver oil, salmon, mackerel, sardines, tuna and egg yolks. Fortified foods would include cow’s milk, plant based milks, breakfast cereals and margarine/spreads. *CYP24A1* disease causing variants lead to nephrocalcinosis and calcium stone formation, and conventional treatments for calcium stones are recommended. These would include maintaining a high fluid intake and avoiding excess dietary sodium. Specific measures would include avoiding dietary vitamin D supplements (in foods and drinks) and avoidance of excessive sunlight exposure [8].

For severe disease phenotypes associated with biallelic genetic changes, treatment with drugs that inhibit P450 enzymes have been tried. Ketoconazole is a non-specific P450 enzyme inhibitor that decreases levels of active vitamin D3 [5, 18]. Low dose fluconazole, via the same mechanisms has also been shown to be effective [15] and alongside lifestyle modifications offers a useful adjunct [7, 20, 21]. Rifampicin is an alternative treatment for severe disease as it induces *CYP3A4*, that contributes to induction of vitamin D metabolites in patients with *CYP24A1* variants [1, 10].

In conclusion, *CYP24A1* disease causing heterozygous variants may be identified in older patients who present with a subclinical course or a late manifestation of disease, suggesting that the phenotypic spectrum associated with these genetic alterations can be broader and more variable than previously recognized. This highlights the importance of considering *CYP24A1* variants not only in younger individuals with classic symptoms, but also in older patients who may exhibit mild or atypical signs, such as unexplained hypercalcemia, nephrocalcinosis, or kidney stones. Early recognition, even in cases with subtle clinical features, can guide appropriate management and prevent potential complications associated with chronic dysregulated calcium metabolism.

## Data Availability

No datasets were generated or analysed during the current study.
